# The Role of Color Doppler Imaging in the Diagnosis of Glaucoma: A Review of the Literature

**DOI:** 10.3390/diagnostics13040588

**Published:** 2023-02-05

**Authors:** Lamprini Banou, Anna Dastiridou, Athanasios Giannoukas, Georgios Kouvelos, Christos Baros, Sofia Androudi

**Affiliations:** 1 Department of Ophthalmology, University of Thessaly, 41334 Larissa, Greece; 2 Department of Vascular Surgery, University of Thessaly, 41334 Larissa, Greece

**Keywords:** Color Doppler Imaging, glaucoma, vascular theory, ocular blood flow

## Abstract

Glaucoma is a progressive optic neuropathy and one of the leading causes of irreversible blindness worldwide. Elevated intraocular pressure (IOP) is the major risk factor for the onset and progression of glaucoma. In addition to elevated IOP, impaired intraocular blood flow is also considered to be involved in the pathogenesis of glaucoma. Various techniques have been used to assess ocular blood flow (OBF), including Color Doppler Imaging (CDI), a technique used in ophthalmology in recent decades. This article reviews the role of CDI in both the diagnosis and effective monitoring of glaucoma progression, presenting the protocol for imaging and its advantages, as well as the limitations of its use. Moreover, it analyzes the pathophysiology of glaucoma, focusing on vascular theory and its role in the onset and progression of the disease.

## 1. Introduction

Glaucoma is a neurodegenerative disease caused by the loss of retinal ganglion cells (RGCs). It is characterized by glaucomatous damage of the optic nerve and characteristic visual field defects [1]. This results in painless and irreversible loss of peripheral vision that can lead to blindness if left untreated. These features often lead to delayed diagnosis and treatment [2]. 

The global prevalence of glaucoma was 3.54% in 2014. The number of glaucoma patients, aged 40–80 years, is estimated to increase by 73%, from 64.3 million in 2013 to 111.8 million in 2040 [3]. According to a 2020 meta-analysis, the global incidence of primary open-angle glaucoma (POAG) is 2.4%, corresponding to 68.56 million patients. [4]. For normal tension glaucoma (NTG) patients, a major subtype of POAG, percentages vary by country of origin, with Asia ranking first [5].

Glaucoma is a multifactorial disease. Although elevated intraocular pressure (IOP) is the major and most well-studied risk factor for the onset and progression of glaucoma, other factors, such as vascular dysregulation, decreased axoplasmic flow within the RGC axons, oxidative stress, and genetic background can also play an important role [6,7]. Optic nerve damage is thought to be the result of a complex interplay, rather than a consequence of a single factor [6].

The vascular theory suggests that glaucoma is a consequence of insufficient intraocular blood flow. Reduced blood flow is now considered an established factor that contributes to the progression of optic nerve damage. The need to search for new pathophysiological mechanisms arose from the fact that increased IOP alone could not explain all glaucoma cases. There are patients who present with IOP within, what is considered, the normal range (NTG), or patients who continue to progress, despite their IOPs being seemingly well controlled with medication, laser, or surgery [6,8,9].

Ocular blood flow (OBF) measurement using Color Doppler Imaging (CDI) has been used in recent decades and has shown reduced velocities in glaucoma patients compared to the normal population [10,11], with many researchers recommending it as a diagnostic tool for the disease. Meanwhile, new studies focus on patients with NTG and the increased role of vascular risk factors in the pathophysiology of the disease [12].

## 2. Anatomical Aspects

Ocular circulation is complex due to the need to provide nutrients to all ocular structures without interfering with the visual pathway [13]. It consists of two different systems that supply the eye: the retinal and the choroidal circulation. The optic nerve (ON) is divided into four areas. The superficial nerve fiber layer is supplied by the inner retinal microcirculation, which are mainly branches of central retinal artery (CRA). The prelaminar region receives its blood supply from the posterior ciliary arteries and the Zinn–Haller circle. The blood supply of the lamina cribrosa is similar to the latter, with the posterior ciliary arteries being the primary sources, either directly or through the Zinn–Haller circle. The retrolaminar region of ON is nourished by the pial plexus and ciliary arteries [14].

## 3. Ocular Perfusion Pressure and Blood Flow Regulation

Blood flow to the eye depends on a pressure gradient, called Ocular Perfusion Pressure (OPP). OPP is defined as the driving force for ocular circulation, calculated by the difference between Mean Arterial Pressure (MAP) and Intraocular Pressure (IOP): [OPP = MAP − IOP] [15,16]. An increase in IOP or a decrease in MAP can lead to a decrease in OPP and therefore to reduced flow into the retina and optic nerve if the vascular resistance is unchanged. The vessels of the ocular circulation, however, are able to adjust their diameter in response to changes in the blood pressure so that the retinal blood flow is kept at a balanced level. This ability is known as ‘autoregulation’ and is responsible for steady OBF in the eye, up to a critical extent, despite changes in OPP [13,16,17]. Once this limit is exceeded, autoregulation ceases to exist [16].

Autoregulation of OBF is described as the ability of an organ to maintain its blood flow despite changes in local vascular parameters, including metabolic, myogenic, and neuronal factors [17]. In the eye, autoregulation is defined as local vasoconstriction or dilation causing reciprocal increase or decrease in vascular resistance, thus maintaining a constant supply of nutrients in response to changes in perfusion pressure [13,15,18]. The autoregulation operates in the retinal microcirculation, which is mostly influenced by local factors. Choroidal circulation is subject to neurogenic control, mainly the sympathetic nervous system [19]. Finally, vascular dysregulation could lead to chronically low or unstable perfusion [15]. Changes in OPP may cause ischemia and oxidative stress, resulting in glaucomatous damage of the optic nerve [10,18,20]. Kim et al., in a 2020 meta-analysis, concluded that reduced OPP in glaucoma patients is an additional risk factor for glaucoma, especially in those with a high baseline IOP [21].

## 4. Pathophysiology of Glaucoma

Over the years, it has been established that glaucoma is a multifactorial disease. Elevated IOP, mainly due to reduced drainage capacity of the aqueous humor, is the major risk factor for the development of glaucoma, and the only, to date, therapeutically modifiable parameter [2]. Lamina cribrosa is the most vulnerable region of the globe, and elevated IOP can lead to compression, deformation, and remodeling of the area resulting in direct mechanical damage of the optic nerve fibers when they pass through the point [2,7]. As the disease progresses, a gradual thinning of the neuroretinal rim occurs, resulting in increased cupping of the optic disc. 

Despite the widespread acceptance of elevated IOP as the predominant factor for the development of glaucoma, it cannot explain cases in which patients present with, what is considered, normal pressure (NTG) or cases in which glaucoma continues to progress despite successful regulation of IOP [8,16]. Interestingly, about 30–40% of patients have normal IOP at the initial diagnosis. Therefore, elevated IOP is an important, but not the only, factor responsible for the damage of the ON [6]. Various additional theories have been proposed as an attempt to explain the etiology of glaucoma, including vascular, genetic, and biochemical theory. 

## 5. Vascular Theory of Glaucoma

The vascular theory of glaucoma suggests that glaucomatous optic neuropathy (GON) is the result of impaired OBF [8,16]. This theory dates back to 1858, when von Jaeger argued that damage to the optic nerve fibers due to increased IOP is mediated by ischemia and not by their compression [13,16]. The mechanisms that lead to abnormal blood flow are not yet fully understood. Flammer et al. suggested that an altered blood flow is the most possible mechanism of damage, through oxidative stress caused by the unstable oxygen supply to optic nerve fibers [13,22]. Insufficient OBF, unstable OPP, oxidative stress, vasospasm, and endothelial dysfunction appear to be the major risk factors for GON [7,18,20]. 

Numerous systemic vascular diseases such as migraine, hypertension or hypotension, peripheral vascular disease, Raynaud syndrome, Primary Vascular Dysfunction (PVD), and Flammer syndrome have been implicated in the development of glaucoma, and especially NTG [20,23,24,25]. Other vascular pathologies that may result in hemodynamic changes in ocular circulation, such as carotid artery stenosis, have raised attention as to whether they constitute an independent causal factor for open-angle glaucoma. Their role remains controversial and more studies need to be done [25,26]. Finally, there is increasing evidence that common neurodegenerative diseases, such as Alzheimer’s disease and vascular dementia, share a common pathophysiologic pathway, as well as shared genetic architecture, with glaucoma [20,27]. Researchers have proposed that abnormalities in retinal circulation may reflect the vascular dysregulation in cerebral microvasculature, especially in patients with NTG [20,28]. 

Many researchers have argued whether reduced ocular blood flow is a primary or a secondary effect. The existence of a secondary element cannot be disputed, as it is obvious that OBF to atrophic tissues is reduced. Compression of the ocular vessels in a progressively deformed lamina cribrosa, secondary to elevated IOP, may lead to reduced OBF [13,16,22]. However, there is a lot of evidence to suggest that flow reduction has many primary elements as well. Initially, reduced blood flow is observed outside the eye, even in the periphery. In addition, further reduction in blood flow leads to further progression of glaucoma damage, and often the reduction is more pronounced in patients with NTG than in patients with ocular hypertension. Finally, optic disc hemorrhages can occur at all stages of the disease, with a higher incidence in patients with NTG [13,22]. All these observations suggest that a significant portion of the reduction in OBF is primary.

## 6. Ocular Blood Flow Measurement

Several techniques have been used to measure intraocular blood flow, but there is no ‘gold-standard’ technique to date. Color Doppler Imaging—CDI, Laser Doppler Flowmetry—LDF, Laser Doppler Velocimetry—LDV, Doppler OCT, and Laser Speckle Flowgraphy—LSFG are the major ones [17,29]. These techniques differ in terms of the parameters they use for calculation of ocular circulation and the measuring range, as well as the depth they can enter [17].

## 7. Color Doppler Imaging 

### 7.1. CDI Principles

**Figure 1:**
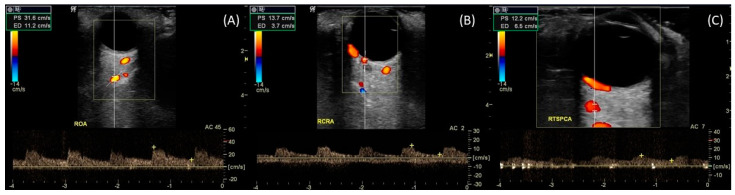
Using the B-scan mode to identify ocular structures. Color Doppler is applied to record the Doppler waveforms: (**A**) Ophthalmic Artery, (**B**) Central Retinal Artery, (**C**) Temporal Short Posterior Ciliary Arteries.

CDI is based on high frequency sound waves and it is a combination of b-scan (gray scale imaging of tissues) along with representation of blood flow by color scale, using the Doppler effect [29]. A linear probe, approximately 7.5 MHz, is used. It is found that the appropriate frequency for measuring blood flow in the intraocular vessels, at a depth of 4–10 cm, ranges from 7.5 to 10 MHz [30]. The probe should not exert pressure on the bulb as it may lead to a false measurement [31]. Most studies are performed with the patient in a supine position with the head at an angle of 30° [29]. The eyelids remain closed, and the patient is asked to look straight ahead [30,32]. The gain must be adjusted correctly to detect low velocities [31]. Proper adjustment of the Pulse Repetition Frequency (PRF) is also required to avoid the phenomenon of false saturation (aliasing) [33]. Finally, it is equally important to choose the right sample volume, so that all the blood flow information throughout the vessel is included in the waveform [31]. The vessels whose velocities are calculated are the OA, CRA, SPCAs, and CRV (Figure 1). The assessment is made by measuring velocities—Peak Systolic Velocity (PSV) and End Diastolic Velocity (EDV)—, and the Resistance Index (RI) in the OA, CRA, and SPCAs (Table 1). PSV and EDV are reported in cm/s and RI is a dimensionless parameter ranging from 0 to 1. CDI is part of the equipment of any large hospital, unlike other flow measurement techniques, which add an extra financial burden to clinics [32].

### 7.2. Doppler Waveform

The Doppler waveform is a three-dimensional representation where the horizontal axis refers to time, and the vertical axis to the Doppler frequency, and the third dimension refers to the amplitude or strength of the Doppler signal, displayed on a multilevel scale of gray. Fourier transform is used for the analysis [30].

### 7.3. Advantages and Limitations

CDI is a non-invasive, easily accessible, cost-efficient technique, used in ophthalmology in recent decades. Moreover, ocular ultrasound has no ionizing radiation exposure, does not require contrast, and is not affected by poor ocular media [34]. It can be performed by either an ophthalmologist, a radiologist, or other operator who has undertaken proper training. It has been used to prevent and diagnose multiple eye conditions including central retinal artery/vein occlusion, ocular ischemic syndrome, glaucoma, diabetic retinopathy, and age-related macular degeneration. In addition, its contribution to the differential diagnosis of intraocular tumors is important, with the assessment of neovascularization [30]. 

To date, an organized database that can be used by operators worldwide is unavailable. Some researchers have formulated mean values for both normal and glaucoma populations, based on existing literature [32,35]. In addition, The Leuven Eye Study is one of the largest databases for ocular blood flow in glaucoma, listing 546 patients [36].

Although CDI is a valuable tool for measuring intraocular velocities, it has limitations. Initially, its use is limited to large intraocular vessels. Due to its inability to measure the diameter of blood vessels in vivo, it can calculate velocities but not blood flow volume [10,29]. Anatomical differences between patients in the density and organization of blood vessels as well as the dependence of the measurements on the placement of the transducer, further complicate this task [29]. Finally, the experience of the examiner has a decisive role in the correct measurement and evaluation of the results, as well as in the improvement of the repeatability of the examination [30,32,37,38]. Founti et al. studied the repeatability of the exam in healthy volunteers among three masked operators and concluded that expert training and execution of a specific protocol for CDI provide highly reproducible results [38]. 

### 7.4. Safety of CDI

Ocular Ultrasound has been used in ophthalmology the past decades, with its use being expanded in recent years. The power of the ultrasound which can be used safely in mammalian tissues was defined by the American Institute of Ultrasound in Medicine (AIUM) [39]. Current safety measures include thermal index (TI) and mechanical index (MI), which reflect the likelihood of ultrasound causing thermal or mechanical (non-thermal) bioeffects, respectively, in the tissue [30,34,39]. Although there has not been a reported incident of adverse bioeffects on humans at diagnostic ultrasound levels, it is known from animal studies that heating of the tissue may occur with ultrasound, so prudent use is advised [39]. The US Food and Drug Administration (FDA) has set a maximum Spatial Peak Temporal Average Intensity (I_SPTA.3_) of 720 mW/cm^2^, MI ≤ 1.9 and TI ≤ 6.0 for diagnostic ultrasound examinations. For ophthalmic diagnostic applications, the FDA recommends exposure levels of I_SPTA.3_ of 50 mW/cm^2^ or less, TI ≤ 1.0, and MI of 0.23 or less, due to the relatively larger absorption of the lens and orbital fat, and the poor perfusion [34,39]. For the eye, the soft-tissue thermal index (TIS) is considered the most suitable TI to use. Although ultrasound devices dedicated to ophthalmic use comply with FDA maximum recommended acoustic output levels, most general-purpose Doppler ultrasounds do not provide an ophthalmic setting. So, it is the user’s responsibility to comply with ophthalmic exposure recommendations. The operator should monitor the TI and MI during the scan to ensure that the values remain within the recommended levels, while employing the ALARA principle (as low as reasonably achievable) [34]. Following the ALARA principle means that we keep total ultrasound exposure as low as reasonably achievable while optimizing diagnostic information in the shortest scanning time possible [30,34,39]. 

## 8. CDI and Glaucoma

As previously discussed, OBF is important in the pathophysiology of glaucoma. Although OBF measurement with existing techniques, including CDI, is not yet considered as a clinical tool to diagnose glaucoma, numerous studies have suggested its use in both the diagnosis and effective monitoring of glaucoma progression. Below we summarize the existing evidence in patients with POAG, NTG, pseudoexfoliation glaucoma (PXG), and angle-closure glaucoma (ACG).

### 8.1. Primary Open-Angle Glaucoma

Several studies have compared glaucoma patients with control groups, demonstrating an abnormally low PSV and/or EDV, along with an elevated RI in glaucoma population. The major studies, available in the literature, are presented in Table 2. Two meta-analyses [10,11], which compiled the literature up to 2015, compared normal population with POAG and NTG, respectively, and suggested CDI as a valuable diagnostic tool for glaucoma. Meng et al. (2013) performed a meta-analysis consisting of 23 studies and a sample of approximately 2000 eyes (POAG/control groups). They concluded that patients with POAG, either with or without treatment, have statistically significant reduced PSV and EDV in the OA, CRA, and SPCAs, as well as statistically significant elevated RI in all vessels. These data are consistent with reduced perfusion of the optic nerve head, and therefore, ischemia. In addition, researchers hypothesize that the thinning of the retinal nerve fiber layer recorded in glaucoma patients is probably the result of insufficient perfusion of the retina as well [10]. In a similar meta-analysis in 2015, Xu et al. included 23 studies with approximately 1700 eyes and compared NTG patients with control groups. Xu et al. reported a statistically significant reduction of PSV and EDV and increase in RI in the OA, CRA, and SPCAs in NTG patients, reinforcing the vascular theory of glaucoma and suggesting that ischemia affects the retina as well as the optic nerve [11].

In more recent studies, the results were in accordance with previous literature, further supporting the presence of impaired OBF in glaucoma (Table 2). The Leuven Eye Study (2016) is one of the largest databases for ocular blood flow in glaucoma, listing 546 patients. The study showed reduced velocities in the CRA and OA in glaucoma patients, with no differences in SPCAs rates between glaucoma and control groups [36]. Kurysheva et al. analyzed data of patients with pre-perimetric glaucoma and concluded that hemodynamic parameters, by measuring flows with CDI, show better reliability for the diagnosis of early glaucoma, compared to structural parameters [40]. Krzyżanowska-Berkowska et al. also concluded that glaucoma patients had impaired blood flow, with reduced velocities in the CRA and OA, in combination with a deformed lamina cribrosa [41].

In addition to OBF, some studies have examined the association of decreased flow with other variables, such as ocular pulse amplitude (OPA) [12,42,43], lack of spontaneous venous pulse [44], morphology of the lamina cribrosa [41], cerebrospinal fluid pressure [45], optic nerve sheath diameter [46], changes of the Doppler waveform [43,47], and disturbed autoregulation of the blood flow [48,49]. 

**Table 2:** Studies comparing Glaucoma versus Control groups.

Study	Objectives	Sample	Results	Conclusions
Meng et al., 2013 [10]	Diagnostic value of CDI in measuring intraocular blood flow in patients with POAG.	23 studies—approx. 2000 eyes (POAG/control groups)	• ↓PSV and ↓EDV in OA, CRA, SPCA • ↑RI in OA, CRA, SPCA	CDI may be used in the diagnosis of POAG patients.
Xu et al., 2015 [11]	Possible diagnostic value of CDI in the assessment of hemodynamic changes in patients with NTG.	23 studies—approx. 1700 eyes (NTG/control groups)	• ↓PSV and ↓EDV in OA, CRA, SPCA • ↑RI in OA and TSPCA	Ischemia is one of the major effects of NTG. Vascular parameters measured by CDI may be used as criteria in the diagnosis of NTG.
Abegão Pinto et al., 2016 [36]	Identify which of the vascular data can be helpful in the clinical practice for screening and disease stratification.	546 patients: 214 POAG, 192 NTG, 140 control groups	Glaucoma patients showed: • ↓intraocular velocities using CDI • higher venous saturation • asymmetries of choroid thickness	The database aims to introduce vascular parameters into daily clinical practice for the diagnosis of glaucoma.
Kurysheva et al., 2017 [40]	Diagnostic value of OBF and choroid thickness parameters in the detection of early glaucoma.	62 eyes: 32 glaucoma, 30 control groups.	• ↓EDV in CRA, SPCA • ↓mean flow in CRA	Hemodynamic parameters, by measuring velocities with CDI, show better reliability for the diagnosis of early glaucoma, compared to the structural parameters.
Eniola et al., 2018 [50]	Comparison of CDI data of CRA and OA in young patients with POAG with normal population.	200 eyes: 100 with POAG 100 control groups.	• ↓PSV and ↓EDV in OA, CRA • ↑RI in OA and CRA	Patients with POAG show reduced velocities and elevated RI of the intraocular vessels compared to the normal population.
Kalayci et al., 2020 [51]	Analyze flow parameters of OA, CRA and ICA using CDI.	145 patients: 35 POAG, 65 NTG and 45 control groups	• ↑RI, PI, PR in OA • ↑RI in CRA	OA PR (peak ratio) and ICA intima-media thickness may be used as diagnostic tools.
Krzyżanowska-Berkowska et al., 2021 [41]	To evaluate association between OBF biomarkers and lamina cribrosa parameters.	211 patients: 70 POAG, 72 glaucoma suspects, 69 control groups.	• ↓PSV in OA, CRA • ↓MFV in OA, CRA	Impaired OBF was found to be associated with deformed lamina cribrosa in glaucoma patients.

CDI: Color Doppler Imaging, POAG: Primary Open Angle Glaucoma, NTG: Normal Tension Glaucoma, OA: Ophthalmic Artery, CRA: Central Retinal Artery, SPCA: Short Posterior Ciliary Arteries, ICA: Internal Carotid Artery, PSV: Peak Systolic Velocity, EDV: End Diastolic Velocity, RI: Resistive Index, PI: Pulsatility Index, PR: Peak Ratio, MFV: Mean Flow Velocity, ↑ increase, ↓ reduction.

### 8.2. NTG versus POAG

Patients with NTG have been associated with a variety of systemic vascular diseases, including migraine, Alzheimer’s disease, primary vascular dysregulation, obstructive sleep apnea syndrome, and Flammer’s syndrome [52]. Many studies have shown reduced blood flow in patients with NTG compared to the normal population [11,36]; however, we have conflicting data regarding the comparison of NTG versus POAG patients. In Table 3, we present all the latest data concerning the differences in velocities among the two groups.

Barbosa-Breda et al. in 2019, using the Leuven Eye Study database, compared 384 patients with POAG and NTG and concluded that patients with NTG show more signs of vascular dysfunction such as peripheral vasospasm, migraine, low MAP [12]. From the ultrasound criteria, what appeared to distinguish the two groups were: a higher RI, higher EDV, and lower Early Systolic Acceleration (ESA) in the CRA in patients with NTG [12]. Higher EDV is probably associated with arterial stiffness or diastolic dysfunction, both signs of systemic vascular dysfunction. Lower ESA is more likely associated with flow disturbance. Similar results were published in [43], with a statistically significant increase in EDV in the CRA in patients with NTG compared to POAG. In addition, many more studies have shown increased EDV, without always reaching statistically significant results (Table 3).

Another parameter that has been found to be a differential criterion for the two groups is PSV in the NSPCAs. Statistically significant reduced flows in patients with NTG compared to patients with POAG were found in two studies [47,53], (Table 3). 

Further research and statistical analysis should be performed to investigate the possible role of Color Doppler Imaging in the differential diagnosis of the two glaucoma groups. The existence of such a differentiation would enhance a variation in both vascular etiological factors, contributing to their different therapeutic approach.

**Table 3:** Comparison of EDV in the CRA and PSV in the NSPCAs in POAG vs. NTG patients.

		EDV CRA		PSV NSPCA	
**Reference**	**POAG/NTG**	**POAG**	**NTG**	**POAG**	**NTG**
[36]	214/192	2.66 ± 0.9 **	2.71 ± 0.98 **	9.39 ± 3.1	9.35 ± 3.3
[42]	17/28	2.4 (0.4)	2.9 (0.9)	10.3 (2.0)	10.1 (3.5)
[43]	74/63	2.63 ± 0.8 *	2.88 ± 0.9 *	9.17 ± 2.8	9.52 ± 3.0
[44]	86/69	2.70 (0.9)	2.96 (1.1)	9.51 (2.9)	9.51 (2.8)
[46]	88/58	2.72 ± 0.8	2.92 ± 1.0	9.68 ± 2.9	9.22 ± 2.7
[47]	102/89	2.75 ± 0.95	2.87 ± 1.08	9.44 ± 2.70 *	8.58 ± 2.69 *
[53]	49/62	2.7 (0.98)	2.8 (0.98)	8.9 (2.3) *	8.2 (2.6) *

[Mean Values (and Standard Deviation/SD) are depicted] * a statistically significant difference between POAG and NTG. ** statistically significant difference only in the multivariate models [12]. POAG: Primary Open Angle Glaucoma, NTG: Normal Tension Glaucoma, CRA: Central Retinal Artery, NSPCA: Nasal Short Posterior Ciliary Arteries, PSV: Peak Systolic Velocity, EDV: End Diastolic Velocity.

### 8.3. Pseudoexfoliation Glaucoma (PXG)

Pseudoexfoliation glaucoma PXG is the most common identifiable cause of secondary open-angle glaucoma [54]. Numerous studies have been carried out to investigate the retrobulbar blood flow changes in patients with PXG. All available studies that meet the criteria published since 2000 are shown in Table 4. There is consistency among studies published so far that a decrease in velocities and an increase in resistivity exists in eyes with PXG compared to control groups [55,56,57,58,59]. However, the existing literature remains controversial regarding CDI findings in eyes with PXG compared to those with POAG [60,61,62,63].

### 8.4. Angle-Closure Glaucoma

There are relatively few studies that have evaluated the hemodynamic parameters in patients with Primary Angle-Closure Glaucoma (PACG). Marjanovic et at. studied OAG versus ACG patients, resulting in increased RI in the OA in OAG as compared to ACG patients, also stating that the degree of OBF disturbance was not related to either the IOP or the visual field loss [64]. On the other hand, Cheng et al. reported that patients with well-controlled PACG may have decreased OBF velocities and increased RI in the CRA and TSPCAs, as compared with healthy subjects. The degree of retrobulbar hemodynamic impairment was well correlated with the degree of glaucomatous visual field damage [65]. Nong and Ninghua investigated the hemodynamic changes of the OA and CRA in PACG and the effects of IOP on the retrobulbar hemodynamics, resulting in increased RI in the CRA of PACG patients. However, the study suggested that elevated IOP was a major cause of the RI elevation in PACG [66].

## 9. Waveform Analysis

**Figure 2:**
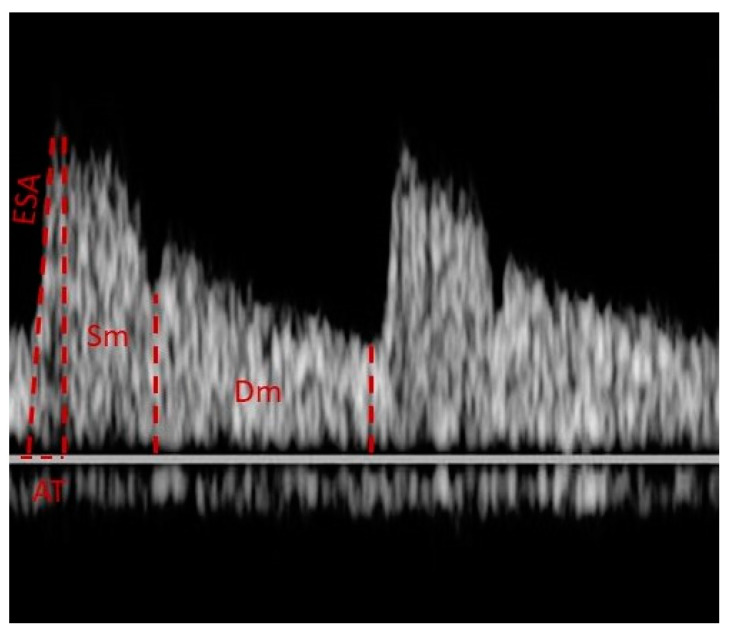
The Doppler waveform of OA. “ESA” corresponds to the inclination of the fastest-moving part of the systolic phase and “AT” to the duration of the above inclination. The “Ratio Sm/Dm” represents the ratio of the mean velocities of the systolic and diastolic phases.

Most studies that use CDI in glaucoma measure OBF by calculating PSV, EDV, and RI. However, other variables have been shown to be useful in the study of glaucoma patients. Early systolic acceleration (ESA) is defined as the inclination of the fastest moving part of the systolic phase and Acceleration Time (AT) as the duration of the above inclination. Another value that can be evaluated is the ratio of the mean velocities of the systolic and diastolic phases (Ratio Sm/Dm) [47], (Figure 2). 

Carichino et al. analyzed 36 images of 36 POAG patients, studying the digitalized waveforms of OA and CRA. They concluded that digital analysis of velocity waveforms can be used to differentiate POAG patients upon demographics or disease severity [67]. Barbosa-Breda et al. concluded that lower ESA in the CRA may distinguish patients with NTG compared to patients with POAG [12]. Pinto et al. studied these variables in 250 people (102 with POAG, 89 with NTG and 59 healthy volunteers). They showed lower ESA as well as lower Sm/Dm ratio in the OA in glaucoma patients. In addition, researchers identified an association between the extent of glaucoma damage and a leftward displacement of the flow, mainly at the expense of a reduction in mean diastolic flow. The data of the study correlate these changes with changes in systemic blood pressure, especially in patients with NTG, suggesting as a causal factor the inability to regulate ocular blood circulation [47]. Pinto et al. concluded that by analyzing the entire Doppler waveform, we can have better results that can be used to compare with other variables, such as ocular pulse amplitude (OPA) [43]. Further studies should be performed to evaluate changes in Doppler waveforms and their role in the development of optic nerve damage.

## 10. CDI as a Tool to Monitor Progression

Numerous studies have been carried out, focusing on CDI as an indicator to monitor glaucoma progression. There is consistency among the studies published that decreased velocities and increased RIs can be used as biomarkers for the progression of glaucoma. The data of the published literature so far are presented in Table 5. Zegadlo et al. conducted a study with 89 patients of different severity which showed that the development of glaucoma damage leads to reduced OBF and elevated RI, simultaneously with the thinning of the RNFL, proposing CDI as a diagnostic tool for the control or treatment of patients at increased risk of a more aggressive optic neuropathy [68]. Kalayci et al. compared a total of 145 patients and found that EDV in the OA was lower and RI in the OA and CRA was higher in both POAG and NTG groups compared to healthy volunteers, suggesting that OBF measurement can be used to determine the severity of the damage and monitor the progression of the disease [51]. Calvo et al. analyzed 262 glaucoma suspects concluding that abnormal retrobulbar blood flow velocities measured by CDI may be a risk factor for conversion to glaucoma. An RI value higher than 0.75 in the ophthalmic artery was associated with the development of glaucoma [69]. Martínez et al. followed 49 POAG patients for 36 months using CDI, concluding that in POAG patients with elevated IOP, RI in the OA and SPCAs may be a reliable indicator of visual field loss [70]. Accordingly, Galassi et al. studied the use of CDI in 44 patients with POAG over a period of 7 years. The results showed reduction in velocities, proportional to the progression of the damage, with progressive visual field loss [69]. 

## 11. Conclusions

CDI may be a valuable tool for both diagnosing and monitoring glaucoma patients. It is a non-invasive, easily accessible, cost-efficient technique with no ionizing radiation exposure, used in ophthalmology in recent decades. Numerous studies have measured reduced velocities and increased resistivity index of the intraocular vessels in glaucoma patients, contributing to the acceptance of the vascular theory of glaucoma as an important etiological factor in the pathophysiology of the disease. Assessing PSV, EDV, and RI in OA, CRA, and SPCAs can provide valuable information for the onset and progression of glaucoma. To date, OBF measurements with existing techniques, including CDI, are mainly used in research protocols. Standardization of the technique and application of a suitable protocol by the operators lead to reliable and reproducible results. An official, organized database that can be used by operators worldwide would be an important step towards the establishment in the clinical practice. Finally, a holistic approach of the vascular factors describing glaucoma patients needs to be established for a more personalized medicine, also focusing on newer antiglaucoma medications that specialize in increasing blood flow to the optic nerve.

## Figures and Tables

**Table 1:** Parameters calculated by CDI.

PSV	Peak Systolic Velocity
EDV	End Diastolic Velocity
RI	Resistive Index, RI = (PSV − EDV)/PSV.
MFV	Mean Flow Velocity
PI	Pulsatility Index, PI = (PSV-EDV)/mean velocity
ESA	Early Systolic Acceleration
AT	Acceleration Time
Sm	Mean velocity of the systolic wave components
Dm	Mean velocity of the diastolic wave components
Sm/Dm	The ratio of average velocities
Vmax	Maximum venous velocity
Vmin	Minimum venous velocity

**Table 4:** CDI studies in eyes with PXG.

	PXG vs. Control Groups	PXG vs. POAG
Kocaturk et al., 2021 [59]	• ↑RI in OA, CRA	-
Dogan et al., 2021 [61]	• ↓PSV, ↓EDV in TSPCAs	No statistical differences
Kocaturk et al., 2016 [58]	• ↓PSV, ↓EDV, ↑RI in OA	-
Sekeroglu et al., 2011 [57]	• ↓PSV, ↓EDV, ↑RI (all vessels)	-
Martinez et al., 2008 [55]	• ↓EDV, ↑RI (all vessels)	-
Martinez et al., 2008 (Eye) [62]	-	•↓PSV, ↓EDV, ↑RI in POAG (vs. PXG)
Galassi et al., 2008 [63]	-	•↓PSV, ↓EDV, ↑RI In PXG (vs. POAG)
Yuksel et al., 2001 [56]	• ↓PSV, ↓EDV, ↑RI (all vessels)	-
Yuksel et al., 2001 [60]	• ↓PSV, ↓EDV, ↑RI (all vessels)	No statistical differences

POAG: Primary Open Angle Glaucoma, PXG: Pseudoexfoliation Glaucoma, CRA: Central Retinal Artery, TSPCAs: Temporal Short Posterior Ciliary Arteries, PSV: Peak Systolic Velocity, EDV: End Diastolic Velocity, RI: Resistive Index.

**Table 5:** CDI as a tool to monitor progression.

Study	Sample	Results	Conclusions
Zegadlo et al., 2021 [68]	89 eyes studied: • 31 preperimetric • 29 early glaucoma • 12 moderate glaucoma • 17 advanced glaucoma	• ↓PSV OA and CRA in advanced than in preperimetric glaucoma • ↑RI CRA in advanced/moderate than in preperimetric glaucoma	CDI may be used as a diagnostic tool for the control or treatment of patients at increased risk of a more aggressive optic neuropathy
Kalayci et al., 2020 [51]	145 patients: • 35 POAG • 65 NTG • 45 control groups	• ↑RI, PI, PR in OA • ↑RI in CRA	OBF measurements may be used to determine the severity of the damage and monitor the progression of the disease
Magureanu et al., 2016 [71]	• 102 patients -202 eyes- with a confirmed diagnosis of GON.	• ↓PSV in CRA was relevant in glaucoma progression	CDI would represent an important diagnosis method, whose results could help adopt more or less aggressive therapeutic measures in conflicted cases
Suprasanna et al., 2014 [72]	• 78 eyes with established POAG -25 with progressive visual field loss and 53 with stable visual field- • 78 control eyes	• ↓EDV in OA and • ↑RI in OA and PCAs in glaucomatous eyes with progressive than with stable visual field loss	OBF appears compromised in eyes with POAG, particularly in those with progressive visual field loss
Jimenez-Aragon F. et al., 2013 [73]	• 71 patients categorized as “Progression” or “No Progression” (5-year follow-up)	Progression group presented: • ↓EDV in OA, CRA • ↑RI in OA, CRA compared to the “No Progression” group	Orbital hemodynamics studied by CDI may represent an important biomarker to discriminate glaucoma patients with higher risk for progression
Calvo et al., 2012 [69]	• 262 glaucoma suspects (48-month follow-up)	• ↑RI > 0.75 in OA was associated with the development of glaucoma	Abnormal OBF velocities measured by CDI may be a risk factor for conversion to glaucoma
Zeitz et al., 2006 [74]	• 114 patients with glaucoma • 40 healthy volunteers	• ↓PSV in CRA • ↓PSV and ↓EDV in SPCAs in patients with progressive glaucoma	Progressive glaucoma is associated with decreased blood flow velocities
Martínez et al., 2005 [70]	• 49 POAG patients (36-month follow-up)	• ↑RI in OA, SPCAs in the eyes that progressed (23 out of 36)	In eyes with POAG and elevated IOP, the RIs of the OA or SPCAs may reliably predict visual field progression
Galassi et al., 2003 [75]	• 44 POAG patients (7-year follow-up)	• ↑RI in OA in patients with visual field loss	CDI variables of OA correlate with the risk of visual field deterioration in patients with POAG

CDI: Color Doppler Imaging, POAG: Primary Open Angle Glaucoma, NTG: Normal Tension Glaucoma, OA: Ophthalmic Artery, CRA: Central Retinal Artery, SPCAs: Short Posterior Ciliary Arteries, ICA: Internal Carotid Artery, PSV: Peak Systolic Velocity, EDV: End Diastolic Velocity, RI: Resistive Index, PI: Pulsatility Index, PR: Peak Ratio, MFV: Mean Flow Velocity, ↑ increase, ↓ reduction.

## Data Availability

Not applicable.
